# A Moral Theory of Public Service Motivation

**DOI:** 10.3389/fpsyg.2020.517763

**Published:** 2020-09-18

**Authors:** Tse-Min Wang, Arjen van Witteloostuijn, Florian Heine

**Affiliations:** ^1^Tilburg School of Economics and Management, Tilburg University, Tilburg, Netherlands; ^2^School of Business and Economics, Vrije Universiteit Amsterdam, Amsterdam, Netherlands; ^3^Antwerp Management School, Faculty of Business and Economics, University of Antwerp, Antwerp, Belgium

**Keywords:** public service motivation (PSM), moral foundation theory, public sector, altruism, prosocial behavior

## Abstract

Morality constructs the relationship between the self and others, providing a sense of appropriateness that facilitates and coordinates social behaviors. We start from Moral Foundation Theory (MFT), and argue that multiple moral domains can shape the meaning of public service and engender Public Service Motivation (PSM). From the lens of cognitive science, we develop a causal map for PSM by understanding the social cognition process underlying PSM, focusing on five innate moralities as the potential antecedents of PSM: Care, Fairness, Authority, Loyalty, and Sanctity. Extending moral domains beyond compassion and justice can provide a disaggregated view of PSM, which may help to identify institutional and cultural variation in the meaning of PSM. We discuss the theoretical implications of synthesizing MFT and PSM literatures, and provide directions for future research that could improve our understanding of PSM.

## Introduction

Alan Kurdi, a 3-year-old Syrian boy, drowned on September 02, 2015 in the Mediterranean Sea when he and his family tried to flee to Europe. Images of his toddler’s lifeless body lying face-down on a Turkish beach made global headlines and reverberated across the world. The image revealed the tragic plight of refugees, and stimulated emotional empathic responses that motivated many people to volunteer and provide physical or material help in the European refugee crisis. One charity helping migrants and refugees, the Migrant Offshore Aid Station, recorded a 15-fold increase in donations within 24 h of the publication of the shocking pictures^1^.

The above story demonstrates the important, sometimes dramatic, role of empathetic emotion in motivating volunteers to act prosocially (Doidge and Sandri, 2019). Sympathy belongs to a set of moral emotions that are “linked to the interests or welfare either of society as a whole or at least of persons other than the judge or agent” (Haidt, 2003, p. 853). Triggered by social stimuli, moral emotions establish a motivational and cognitive state in which there is an increased tendency to engage in prosocial actions. Therefore, motivation to perform public service can be seen as an emotional goal system that responds to social stimuli throughout life events and in institutional environments. In this article, we aim to reposition moral emotions inside the theory of Public Service Motivation (PSM) by examining the social cognition process underlying PSM.

Public Service Motivation (PSM) is a prominent concept within the domain of Public Administration. PSM theory was developed in an attempt by public administration scholars to challenge the rational-choice perspectives on bureaucratic behavior, which assume a rational and self-interested agent who pursues personal gains such as reputation, power, and monetary rewards. However, goals are usually less specified in public organizations, and performance is more difficult to measure and link to external rewards, so the variation in behavior is more reflective of variation of individual differences than incentive structures (Shamir, 1991). Therefore, PSM emphasizes the important role of self-determined motivation such as moral obligation, intrinsic motivation, and affection in explaining work behavior and job performance in public organizations.

In the last decade or so, studies have extended the concept of PSM to explain a predisposition or attitude to help others and enhance the well-being of society, linking PSM to activities such as volunteering or donating (Perry et al., 2008; Clerkin et al., 2009; Coursey et al., 2011; Lee, 2012). Accordingly, Vandenabeele (2007) defines PSM as a set of value-laden behavioral determinants: The beliefs, values, and attitudes that transcend individual and organizational interests, motivating individuals to think about what is appropriate for society and to act accordingly. In other words, PSM relates to a sense of public morality that responds to institutional stimuli, and which motivates individuals to regulate selfishness (Staats, 1988).

The measurement scale of PSM has been first developed by Perry (1996), and has been revised through a cross-culture survey study into a validated international scale (Kim et al., 2012). PSM is a multidimensional construct with four types of motives: Compassion, Attraction to Public Service, Commitment to Public Values, and Self-Sacrifice. Compassion is an individual’s affective commitment to concern for the welfare of others or society at large. It entails love and a desire to protect people from distress. Attraction to Public Service refers to an instrumental motive driven by the internal satisfaction or enjoyment from serving the public. Commitment to Public Values reflects a norm-based motive to fulfill societal obligations and pursue public values. Self-sacrifice is a prosocial tendency to make personal sacrifice in order to contribute to the well-being of others or society at large. Based on these four dimensions, the greater the level of one’s PSM, the more likely one is to act beyond monetary or reputational benefits, and to engage in behavior that serves the public.

Prior work identified antecedents of PSM such as individual characteristics, sociohistorical contexts, and organizational influences (Perry, 1997; Brewer et al., 2000), but causal mechanisms underlying PSM are still underdeveloped and much less investigated (Bozeman and Su, 2015). Only a few empirical studies investigate the role of basic psychological needs in explaining the motives to serve the public interest. Further work is needed to understand the origin of PSM, and to develop a comprehensive theory of PSM that can explain cultural and institutional differences (Perry and Vandenabeele, 2015). Furthermore, PSM has long been theorized as a sense of public morality grounded in the public sector, but whether PSM is a genetically predisposed trait or a learned attitude remains contested in the PSM literature, and scholars urge for more work on a causal map for PSM (Bozeman and Su, 2015).

The current study offers a model to shed some light on the psychological orgin of PSM, which will co-define the future empirical agenda. The psychological dispositions to help others and act accordingly are inherent to all human beings, and PSM is the result of a mental representation that links these innate dispositions with stimuli grounded in the public institutions to engender a logic of appropriateness (i.e., “what behavior is appropriate given who I am and what I want to be”). Perry’s (1996) four dimensions of PSM categorize the integrated mental representation, which includes beliefs, attitudes, and experiences about public service from long-term memory. In other words, performing public service becomes ‘moralized’ through the recurrent interaction between innate human moralities and relevant stimuli from the institutional environment, which engenders a feeling of obligation and affective commitment.

As said, the current study contributes to this literature by applying Moral Foundation Theory (MFT; Graham et al., 2013), and insights from relevant neurobiological studies, to explore the role of innate moralities as potential antecedents of PSM. MFT and associated empirical work have developed validated measures of the moral profiles of individuals. MFT postulates that humans are motivated to suppress selfishness by various combinations of cultural traits, referred to as moral foundations (MFs), which are innate, modular, and irreducible. In line with this theory, we argue that people feel motivated to provide public service because moral foundations trigger a socially and institutionally competent person to regulate selfishness and collaborate with others by eliciting PSM-relevant beliefs, attitudes, and memories. This logic implies that social stimuli that emerge throughout life events and in institutional environments contribute to the onset and recurrence of PSM. Furthermore, according to MFT, this motivational influence is both constructed and constrained by a restricted number of five moral foundations: Care, Fairness, Authority, Loyalty, and Sanctity. Additionally, after describing and illustrating our theory, we will suggest a future empirical research agenda.

Our article is organized as follows. First, we summarize the existing literature on the relationship between moralities and PSM. Second, we introduce Moral Foundation Theory and discuss its relevance to PSM theories. Third, we incorporate insights from neurobiology to present the social cognition process of PSM, explaining how innate moral foundations shape prosocial motivation to affect social behavior. Finally, we elaborate the process of moralizing public service for each moral foundation, and explore its behavioral implications and boundary conditions. We conclude with a brief discussion of our contribution, and reflect upon a few promising research opportunities that may feed into a systematic empirical inquiry of the fundamental moral roots of serving the public.

## Moralities and PSM

Perry and Wise (1990) define PSM as a pluralistic construct to understand the human motivation to serve the interests of society, and to explain individual behavior in public organizations, such as job performance and satisfaction. A series of studies have demonstrated that PSM is a general, altruistic motivation to serve the public that is not exclusively grounded in public institutions (Rainey and Steinbauer, 1999; Liu et al., 2008; Perry and Hondeghem, 2008). PSM is a mix of motives that drive an individual – regardless of being employed in the public sector or not – to take social responsibility, suppress selfishness, and benefit society. For instance, PSM has been associated with a variety of prosocial behaviors, such as volunteering and donating time or blood (Houston, 2006; Coursey et al., 2008; Perry et al., 2008; Clerkin et al., 2009; Lee, 2012; Piatak and Holt, 2020). The relationship between PSM and observed prosocial behavior is also found in laboratory and field experiments: People with higher PSM are more altruistic, egalitarian, cooperative, and trustworthy, and are more likely to undertake altruistic punishment to uphold social justice (Esteve et al., 2015, 2016; Tepe, 2016; Tepe and Vanhuysse, 2017; Prokop and Tepe, 2020).

Research has identified a variety of PSM antecedents, such as individual sociohistorical characteristics and organizational influences (Perry, 1997; Moynihan and Pandey, 2007; Perry et al., 2008). Perry (1997) finds that individual formative experiences such as parenting, religion, schooling, and profession are significant for the development of PSM. He postulates that moral development could play a role in socializing individuals through social and interpersonal interactions. However, research on the antecedents of PSM has mainly focused on institutions and environments that interact with the basic psychological needs of each individual (Taylor, 2007; Kim and Vandenabeele, 2010), and only a few studies have examined basic psychological needs as fundamental antecedents of PSM. Van Witteloostuijn et al. (2016) constitute one of the few exceptions to investigate the role of fundamental personal traits in explaining PSM. A fuller understanding of basic psychological needs could help establish whether PSM is a stable trait or a dynamic state (Bozeman and Su, 2015), and to explain the differences in behavioral and organizational implications of PSM (Van Witteloostuijn et al., 2016), as well as in the meaning and scaling of PSM dimensions across different cultures and languages (Kim et al., 2012).

Perry (2000) argues that moral convictions, beliefs, and ideologies play essential roles as social institutions determining people’s motivation and behavior in the public sector. Morality is an expression of the relationship between the self and others (Staub, 1993). It makes up an individual’s identity and values that help individuals distinguish the difference between right and wrong, and create corresponding obligations and motivations. PSM can be interpreted as a sense of public morality (Staats, 1988), rooted in a logic of appropriateness, being defined as a set of belief about what is right or wrong according to who “others and I think I am.” Such morality is characterized by institutional values and transmitted to individuals through identity and beliefs (Vandenabeele, 2007). Moral values and identity make up an individual’s self-concept and engender a logic of appropriateness, which has motivational consequences in performing public service (Perry, 2000). Studies have shown that moral values and worldviews could affect individual motivation, and shape collaborative and ethical behavior in the public sector (Perry et al., 2008; Conner et al., 2015; Stazyk and Davis, 2015).

Moral values provide attitudes, beliefs, and norms about the relationship between the self and the social world, helping people to suppress the self-interest and to pursue the interest of the common good instead. Likewise, PSM is rooted in the notions of the common good, encouraging public employees to act out of compassion, sacrifice personal interests, and endorse public values. PSM-relevant beliefs and norms are inherent in and connected with the moral high road, an ethical approach that relies on personal integrity and moral intuition (Stazyk and Davis, 2015). This important role of morality in engendering PSM does not rule out learning and internationalization of laws, institutional rules, and professional standards. High-PSM individuals can associate institutional values with their internal moral systems through socialization, environmental reinforcement, and value congruence (Wright and Pandey, 2008; Stazyk and Davis, 2015).

In sum, morality makes up an individual’s self-concept by providing a logic of appropriateness about the social relationships between the self and others, als in relation to the public domain. Individuals with high PSM can be seen as “moral exemplars” who pursue their moral goals to achieve a life characterized by deep integration of self and public morality (Perry, 2000; Perry et al., 2008). In the following sections, we draw from Moral Foundation Theory to explore this alleged moral content of PSM, and to understand how individuals construct their moral identity.

## Moral Foundation Theory

Traditional approaches in moral psychology research often treat moral judgment as a rational and deliberative process (Kohlberg, 1969; Rest, 1986). The cognitive-developmental approach assumes a stage theory where individual moral cognition progresses, and becomes more sophisticated, through a series of development stages (Kohlberg, 1969). Rest (1986) portrays individuals’ moral decision-making as a four-component process: awareness, judgment, motivation, and behavior. Empirical research has identified several individual and contextual influences on these four processes, including cognitive biases, identity, leadership, and reward systems (Treviño et al., 2006). However, Kohlberg’s theory has been criticized for its reliance on a limited set within moral philosophy, particularly liberal ideals (Hogan et al., 1978; Shweder and Kohiberg, 1994; Graham et al., 2011), and for its assumption that moral deliberative reasoning is the basis of moral judgments and behaviors (Haidt, 2001). In Kohlberg’s theory, morality is centered on the protection of individuals, so conservative ideas are not acknowledged to be moral principles, such as loyalty to the ingroup, respect for the superior, and avoidance of spiritual pollution (Haidt and Graham, 2007).

Recent approaches consider moral behavior to be strongly influenced by intuitions and emotions. Haidt’s (2001) social intuitionist model of moral judgment maintains that “moral judgment is generally the result of quick, automatic evaluations (intuitions)” (p. 814). Moral judgment is innate, intuitive, and emotional so that our moral mind is organized in advance of experience, and prepared to learn values, norms, and behaviors related to social problems, which explains why individuals often feel, physically and emotionally, self-righteous about moral propositions (Haidt, 2001; Haidt and Joseph, 2004). This approach has opened the door to reexamine the functional content of human intuitive responses regarding moral issues, and to expand the moral domain beyond altruism and fairness concerns. Graham et al. (2011) develop a social intuitionist model, known as Moral Foundation Theory (MFT), to investigate the plurality of moral intuitions and to broaden the moral domain that matches the anthropological accounts of morality. Moral foundations are an affective, evolutionary response of human ancestors facing a diverse set of longstanding adaptive challenges to organize social lives (Keltner and Gross, 1999; Keltner et al., 2006; Haidt, 2007). Organized in advance of experience and prepared to learn values, moral foundations enable humans to write and interpret moral codes that guide patterns of behavior across different cultures and societies.

Moral Foundation Theory emphasizes the affective primacy of moral judgment. Innate moral intuitions enable humans to solve collective action problems by making automatic, quick, and affective reactions to stimuli (Haidt, 2007). Higher-level cognitive thinking is preceded and stimulated by affective reactions that motivate people to adopt approach or avoidance strategies (Zajonc, 1980). This nativist perspective does not preclude cultural learning: Moral foundations are not finished moralities, but only constrain how moral codes can evolve. Social environments are important in the process of moral development: different religions, cultures, and institutions have coevolved with complex practices, stories, and norms for people to find their moral mind and develop their social knowledge. Evolution has shaped brains that are prepared to learn patterns of the social world, and innate psychological mechanisms have coevolved with cultural institutions and practices in a long history of humankind (Gifford, 2008). This intuitionist perspective has been supported by psychological experiments and neuroscience evidence (see, for example, Greene et al., 2001; Greene and Haidt, 2002; Cushman et al., 2006; Luo et al., 2006; Gore and Sadler-Smith, 2011; Sinclair, 2011; Everett et al., 2016), and has been applied in psychology, anthropology, behavioral economics, cognitive science, and organization studies (e.g., Greene and Haidt, 2002; Weaver et al., 2014; Clark et al., 2017; Ellemers et al., 2019; Enke, 2019).

Moral Foundation Theory takes a pluralistic morality approach, expanding the previous narrow concern for justice, welfare, and rights to the duty of social role fulfillment (Graham et al., 2011, 2013). MFT delineates the moral mind into five content domains: Care/Harm, Fairness/Cheating, Authority/Subversion, Loyalty/Betrayal, and Sanctity/Degradation. These five dimensions can be collapsed into two larger categories: individualizing and binding foundations. Care and Fairness are individualizing foundations, as their focus is on an individual. Loyalty, Authority, and Sanctity make up the binding foundations, as they bind people together by promoting duty, order, and cohesion. Binding foundations are related to the domain of human morality because they serve the social functions of limiting autonomy and self-expression for the good of social communities such as families, teams, and nations (Graham and Haidt, 2010). Cross-cultural research on moral codes has revealed that various societies rely on different interpersonal moral codes to regulate behavior: collectivistic cultures such as India and Japan emphasize social harmony and a duty-based interpersonal moral code, while individualistic cultures such as the United Kingdom and the United States stress autonomous voluntarism and an individually oriented moral code (Markus and Kitayama, 1991; Miller, 1994; Singelis et al., 1995; Akkermans et al., 2010).

Different institutions – from private firms and government agencies to cultures and societies – can employ a specific configuration of moral foundations to shape diverse social relationships, political ideologies, and actual behaviors. The difference is not just cultural, between modern and traditional societies, but individual: Even within Western societies, liberals prioritize individualizing foundations over the binding ones in their moral judgments, whereas conservatives apply individualizing and binding foundations equally (Graham et al., 2009). Individual differences in moral foundations have been found to have effects on political identity, donation behavior, and attitudes toward public issues such as climate change and punitive policies (Dawson and Tyson, 2012; Winterich et al., 2012; Dickinson et al., 2016). Recently, management scholars started to use MFT as a framework to investigate organizational behavior, prosocial behavior, and ethical leadership (Winterich et al., 2012; Fehr et al., 2015; Egorov et al., 2017; Jancenelle and Javalgi, 2018). In sum, moral foundations could take a significant role in predicting motives toward social behavior and collective action.

MFT’s biggest advantage is its pluralistic and modular approach. MFT is aimed to provide a positive, descriptive investigation of human morality across cultures, and its modular approach enables researches to refine and extend the moral domain in the face of new evidence (Graham et al., 2013). Graham et al. (2013) offer examples of moral judgment that cannot be produced by a single mental process. Harm-based moral monism is not sufficient to describe moralized values in non-Western societies (Graham and Haidt, 2010). For example, Buchtel et al. (2015) show that Chinese, compared to Western people, are less likely to associate immorality tightly to harm, even in the case of killing where the harm is intentionally inflicted upon a suffering person. By incorporating insights from MFT, we can explore the moral variation of PSM and provide a dis-aggregated view of PSM dimensions, improving our understanding of the mechanisms behind various behavioral relationships (Perry and Vandenabeele, 2015).

Another advantage of MFT is its emphasis on moral emotion, such as pity, guilt, pride, or disgust, and incorporation of cognitive science. Moral emotions are strong motivational states that link perception of social stimuli to social behaviors by constructing a mental representation of oneself as situated within a community or society (Adolphs, 2003, 2009; Tangney et al., 2007). Moral emotions not only construct how we feel about social events, but also motivate us to act accordingly; they function to suppress self-interest and trigger altruistic helping and punishment in the long-term interest of a social group (Adolphs, 2009). In line with MFT, PSM is a contextually dependent disposition that motivates individuals to act in ways that are consistent with their moral self-concept, including their internal value system and cultural identity (Perry, 2000). Therefore, we propose that PSM relies on moral foundations to associate the self-concept with institutional and other contextual stimuli, activating public employees’ motivation to perform public services. We also argue that specific moral foundations are associated with certain aspects of PSM, and thereby may influence social behavior differently. In the following section, we will elaborate on this cognition process and redefine the PSM constructs through the lens of neuroscience to emphasize the important role of moral intuition in engendering PSM.

## Social Cognition Processes and PSM

**FIGURE 1:**
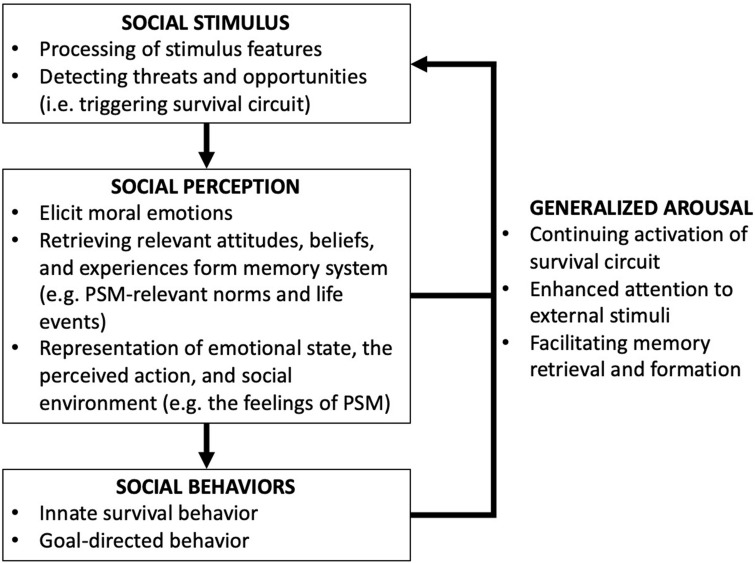
Social cognition process (synthesizing from Adolphs, 2001, 2003; LeDoux, 2012).

Social cognition processes rely on neural mechanisms for perceiving, recognizing, and evaluating stimuli, which together provide information required to construct motivation, emotion, and cognition regarding the social environment (Adolphs, 2001). Moll et al. (2005) suggest that moral behaviors are products of the integration of social perception, contextual knowledge, and basic emotional states. Figure 1 summarizes the social cognition process regarding social behaviors. Triggered by a stimulus, perception first provides relevant information to cognition, and cognition responds to stimuli by guiding automatic or controlled behavior. Moral judgments are mostly direct products of emotional processes (Haidt, 2001; Nichols, 2002; van den Bos, 2003), but reasoning still plays a role in moral behavior as well (Greene et al., 2001; Haidt, 2001; Moll et al., 2003; De Schrijver, 2009; Forbes and Grafman, 2010). However, we often use reasoning to justify our automatic moral intuitions (*post hoc* justifications) or persuade others (reasoned persuasion) (Haidt, 2001).

In the stage of social perception, neural functions and circuits related to survival (survival circuits) participate in processing socially relevant stimuli, detecting opportunities and threats, and modulating behavioral responses when facing particular kinds of challenges and opportunities (LeDoux, 2012). In this stage, the amygdala plays a key role in evaluating morally salient actions, attributing emotional or social value to the stimuli and linking perceptual representations to cognition (Adolphs, 2001; Shenhav and Greene, 2014). In the stage of cognition, specific emotions, memories, beliefs, and motivations that are relevant to the perceptual representation are elicited and integrated to construct higher-order representations of the social environment that can guide social behavior. Besides innate survival behaviors, goal-directed actions that are associated or reinforced through life experiences can then be stimulated in the ventromedial prefrontal cortex, which collects goal-relevant affective information and forms integrative representations that guide behaviors (Shenhav and Greene, 2014).

Overall, the detection of a threat or an opportunity by survival circuits can have three behavioral consequences: (1) the elicitation of hard-wired/innate behavioral reactions; (2) the performance and learning of goal-directed actions through association and reinforcement; and (3) the generalized arousal in which a feedback loop is established to facilitate the continuing activation of survival circuits, to enhance attention to external stimuli, and to stimulate memory retrieval and formation (LeDoux, 2012). The overall result establishes a state of arousal in which brain resources are coordinated and monopolized to cope with threats or opportunities.

Integrating these interdisciplinary insights, we argue that PSM is a cognitive process to construct high-order representations of the social environment that enable individuals to regulate selfishness and serve the interest of a larger community in the public sector. Social behavior is tightly coupled to and heavily regulated by emotion, and moral emotions have been found to serve an essential and privileged role in guiding altruistic and punitive behaviors (Adolphs, 2003). In the stage of social perception, moral foundations play an important role in detecting social opportunities and threats, and in eliciting automatic and emotional responses that stimulate the mental construction process. Therefore, eliciting prosocial motivation such as PSM involves a neural mechanism in which innately specified moral foundations are associated with path-dependent social experiences and recurring social stimuli, engendering the feelings of compassion, commitment, and meaningfulness regarding public service.

Once moral foundations are triggered and stimulated to a point of awareness, relevant beliefs, values, and memories are retrieved to construct high-order representations of the social environment that can create a logic of appropriateness, and drive pro-social and other desirable behavior in public institutions. PSM is “grounded primarily or uniquely in public institutions and organizations” (Perry and Wise, 1990, p. 368) because stimuli generated in and around public institutions are moralized through the association with the extant triggers of moral foundations. Besides, a dual process of social cognition implies that PSM is multi-dimensional and entails affective, normative, and rational motives: Automatic and controlled processes work in tandem to construct a motivated state to cope with opportunities and challenges. Together with triggered moral emotions, PSM-relevant beliefs and lived experiences are retrieved and evaluated, and become the ingredients of a motivated state that helps individuals to understand the relationship of the self with others and with the environment, engendering the sense of public morality or the prosocial identity.

The ability to construct and adopt high-order representations of the social environment that can motivate individuals to serve the public interest is quite flexible, even though this capacity involves an individual psychological makeup that is innate and not fully immutable (Adolphs, 1999). Through the lens of social cognition, the reasons for such flexibility are twofold. First, since innate morality is diverse, different institutions can utilize different configurations of moral foundations to provide individuals with different codes of conduct, social identities, and motivational vocabularies. As a result, public service can be associated with different sets of moral foundations in different cultures and public organizations. Second, Perry (1996)’s four dimensions of PSM can be regarded as the conscious feelings that individuals construct to represent their state of emotion and arousal. Such mental construction involves matching the emotional state with long-term memory stories, experiences, and languages as reinforcers of behavior (LeDoux, 2012)^2^. Hence, individuals can hold different conceptions of PSM (Brewer et al., 2000; Schott et al., 2015), associating different lived experiences and life events with innate morality.

In the following two subsections, we will apply the social cognition process to investigate how each MF can be associated with PSM-relevant beliefs and attitudes to moralize public service, and to explore behavioral implications in the existing PSM literature. Table 1 summarizes our propositions by showing how each moral foundation is triggered to subsequently construct PSM to effect certain types of prosocial behaviors.

**TABLE 1:** The social cognition process of Public Service Motivation (PSM).

		Care	Fairness	Authority	Loyalty	Sanctity
**Social stimulus**	**Triggers**	Suffering, distress, or neediness	Cooperation and defection	Signs of ranks and status	Signs of ingroup and outgroup	Degradation, corruption, piety
	**Opportunity or threat to**	Wellbeing of others	Social justice	Social stability	Group cohesion and security	Spirituality
**Social perception**	**Moral Emotions**	Compassion, anger	Gratitude, anger, guilt	Pride, respect, fear	Group pride, shame, anger	Disgust, elevation
	**Locus of attention and belief elicitation**	Feelings and perspectives of others	Welfare distribution, trustworthiness	Division of roles and responsibilities	Membership boundaries, group identity	Meaning and connection to higher purpose
	**Logic of appropriateness**	Guardian of people in need and distress	Guardian of the underprovided and mistreated	Guardian of rules and institutions	Guardian of the community	Guardian of transcendent purposes
	**Relevant PSM concepts**	Compassion, benevolence, and kindness	Equality, equity, and individual rights	The sense of duty, professionalism	Patriotism, social security, citizenship	Temperance, a calling of public service
**Social behavior**	**PSM behavior**	Helping behavior	Reciprocal cooperation, altruistic punishment	Bureaucratic behavior	Citizenship behavior, group-based altruism	Self-transcendence behavior
	**Evidence in practice**	Social volunteering, knowledge sharing, collaboration	Administrative equality, collaboration	Rule-abiding, leadership/fellowship, civic (dis-)obedience	Social security service, community development	Pro-environment behavior, self-restraining

## Individualizing Foundations and PSM

Individualizing foundations, comprising Care and Fairness, are primarily concerned with individual rights, freedom, and autonomy. People try to recognize kindness, promote reciprocity, and avoid unfair defection. *Care* involves an ability to feel the pain of others, and underlies virtues of kindness and gentleness. It responds to the adaptive challenges of taking care of vulnerable offspring and promoting other-regarding prosocial helping; compassionate individuals are considered to be more attractive in mate selection, and desirable in cooperative relations (kinship or friendship). It helps individuals to participate in social relationships by identifying with the welfare of others and recognizing kindness.

The Compassion component of PSM is an affective motive to identify others’ wellbeing and help those in need. Compassion and care are interchangeable terms that refer to other-orientedness, along with sympathy, tenderness, and kindness (Graham et al., 2009; Goetz et al., 2010). As stated at the beginning of the article, the death of Alan Kurdi increased attention to the suffering of others and stimulated empathetic concern that motivated many people to provide humanitarian support, demonstrating the important role of Care in motiving people to provide public service. Likewise, Bagozzi and Moore (1994) present evidence that public service advertisement can induce prosocial behavior by stimulating emotions and sympathies toward the suffering of others.

The suffering and neediness of others act as social stimuli that trigger the Care foundation to increase attention to others’ wellbeing (perspective-taking), and elicit relevant beliefs and attitudes from past memories. If public service has been moralized (conditioned with Care by the social environment) as an appropriate response to alleviate the suffering or increase the wellbeing of others, then it is more likely to establish generalized arousal toward public service. In this case, information about the Care foundation (the survival circuit), observed feelings (sensory information), and beliefs and attitudes toward the consequences of intervention (mnemonic information) then integrate to construct the higher-order representations that we label as the feeling of Compassion. As a result, Care-driven PSM constructs a Samaritan logic of appropriateness: they see themselves as guardians of the people in distress and need, and they perform public service in order to increase the wellbeing of others (Brewer et al., 2000).

Because of the increased attention to others’ individual wellbeing, the Care foundation is more likely to be associated with helping behaviors such as sharing, comforting, rescuing and helping (Underwood and Moore, 1982; Sibicky et al., 1995; Doris et al., 2017). Social volunteering and donation to charity, as the opening story in this article does illustrate, are two prominent examples. Other examples found in practice are collaboration and knowledge sharing: Affective expressions embedded in compassion are found to help to diffuse trust, which is essential for collaboration and knowledge sharing in public organizations (Amayah, 2013; Eldor, 2017).

Proposition 1: Triggered by the suffering and neediness of others, Care moralizes public service through increased attention to others’ wellbeing, elicits the affectation of compassion that values others’ wellbeing, and stimulates helping behaviors.

*Fairness* is the result of the evolutionary process of reciprocal altruism. People are sensitive to signs of cooperation and cheating, and tend to play “tit for tat” with emotions that motivate them to sacrifice their material well-being (Graham et al., 2013). Fairness enables individuals to recognize the social relationship with different others, and to appreciate the values of other individuals (Cosmides and Tooby, 1989). Signs of cooperation and defection, such as other’s kindness or cheating, trigger Fairness and its relevant moral emotions, such as guilt after cheating others, anger at unfair treatment, and gratitude for other’s kindness. Fairness increases attention to the cost and benefit of an action, and elicits beliefs about its implication for social welfare or long-term cooperation.

The normative component of PSM entails public values such as equality and concern for future generations (Kim et al., 2012). These public values can trigger Fairness, and become key ingredients that stimulate arousal of commitment in social justice, equality, and individual rights (Cropanzano et al., 2011). Fairness-driven PSM constructs a humanitarian logic of appropriateness: those driven by Fairness see themselves as guardians of the underprivileged and mistreated, and perform public service in order to uphold or restore social justice (Brewer et al., 2000). For instance, Pedersen et al. (2017) find that citizens with higher PSM are more concerned about administrative equality.

Behavioral economics has extensively studied people’s fairness concerns, reporting overwhelming experimental evidence that concerns for fairness and reciprocity strongly motivate a majority of people to exhibit reciprocal cooperation and altruistic punishment (Rabin, 1993; Fehr and Schmidt, 2006). For instance, Clark et al. (2017) find that people who endorse the individualizing foundations over the binding ones can display a higher level of cooperative behavior in prisoner’s dilemma and trust games. Therefore, Fairness-driven PSM is more likely to stimulate reciprocal cooperation and altruistic punishment (Esteve et al., 2015; Prokop and Tepe, 2020).

Proposition 2: Triggered by cooperation and defection, Fairness moralizes public service through increased attention to welfare distribution, elicits the sense of commitment in public values such as social justice and equality, and stimulates reciprocal cooperation and altruistic punishment.

## Binding Foundations and PSM

Binding foundations, comprising Authority, Loyalty, and Sanctity, are focused on binding together individuals into a cohesive unit. Binding foundations emphasize role steadiness, duty, and self-control to build a well-ordered stable community. At first glance, binding foundations may look at odds with the PSM-relevant values, which include equality, human rights, and democracy (Kim and Vandenabeele, 2010). These three foundations are presented particularly to understand human nature, and to explain the religiosity and social tradition beyond Western, educated, industrialized, rich, and democratic (WEIRD) societies (Graham and Haidt, 2010; Henrich et al., 2010). Fukuyama (2018) argues that most people’s inner self is not based on individuality and autonomy, but “actually constituted by their relationship with other people, and by the norms and expectations that those others provide” (Chapter 6, para. 15). Therefore, MFT’s pluralistic approach can explore moral variations across cultures and institutions regarding the content of PSM: binding foundations provide psychological imperatives for individuals to develop collective identities that could be defined by tradition, nation, or religion. In line with this perspective, Brewer et al. (2000) show that people can be motivated to perform public service with diverse causes beyond compassion and justice: prestigious work and a love of country can stimulate PSM as well.

*Authority* underlies virtues of leadership and followership, including deference to legitimate authority and respect for traditions. Authority was initially a response to the adaptive challenges of building a hierarchical society to coordinate the associated large-scale division of labor. Authority values the recognition of status, the sense of obligation for subordinates to comply, and the sense of legitimacy and desirability for social hierarchy. However, human hierarchies depend not merely on dominance (the threat of force), but much more strongly on freely conferred deference (Henrich and Gil-White, 2001). Today, the efficiency of large modern nation-states relies on rational/legal authority. Authority enables citizens to grant legitimacy and confer deference to public institutions such as judicial courts and police departments (Haidt and Graham, 2006; Lipsky, 2010, p. 57).

Authority involves a psychological ability to improve the efficiency of social learning and cultural transmission by identifying and preferentially imitating role models who are likely, or hopefully, to be skilled and knowledgeable (Henrich and Gil-White, 2001). By creating roles and duties, Authority helps individuals to recognize their leaders as role models, and to internalize the values that their supervisors endorse and exhibit. A classic example of the Authority foundation is Plato’s Republic: The guardians derive their authority from their superior wisdom and virtues, and the auxiliaries take civic courage/duty to enforce the convictions of the goodness (but see Popper, 1957). Recognition of superiority and self-esteem are the key psychological imperatives that motivate the guardian or warrior class to risk their lives and defend the larger community^3^.

Triggered by signs of rank and status, Authority fulfills the psychological needs of being honored for virtues and competences, and moralizes public service by eliciting moral emotions such as pride and respect, and instilling a sense of professional and civic duty (or relational psychological contracts; see Rousseau, 1995) to take official or civic responsibility. It elicits beliefs and behaviors as to what is expected with regard to social roles and duties, and constructs a bureaucratic logic of appropriateness: public officials see themselves as guardians of the society with superior virtues and thus a privilege to enforce the law, and the general public see themselves as citizens who comply with the law in exchange for social stability.

Authority-driven PSM engenders a sense of duty and elicits feelings of pride and respect in performing public service, making a public service career not merely attractive, but also professional: Public servants are obligated to take higher ethical standards, and to accept higher expectations from citizens (Brewer et al., 2000). Lipsky (2010, p. 57) observed that compliance in most street-level bureaucracies arises not merely from fear of punishment, but also from the superior status and legitimacy that citizens grant to the authority in line with the expectation of high ethical standards and professional knowledge. For instance, defenders speak to judges respectfully with the expectation of a fair judicial treatment. Within public organizations, hierarchical authority, scribed duties, and formal rules engender the sense of meticulous respect for protocol, which mitigates probity hazards of public sector transactions (e.g., misinformation, power abuse, and regulatory capture) and ensures the legitimacy of public institutions (for the transcation cost intepretation of Authority, see Williamson, 1999). In short, Authority-driven PSM can stimulate behaviors that are consistent with Weberian bureaucratic values, including accountability, rule abidance, and due process, which have been shown to be positively associated with PSM and the commitment to public values (Andersen et al., 2013).

Proposition 3: Triggered by signs of rank and status, Authority moralizes public service through increased attention to the division of roles and responsibilities, elicits the sense of professional or civic duty of public service, and stimulates bureaucratic behavior.

*Loyalty* promotes self-sacrifice for the in-group, and vigilance against traitors and the out-group. It triggers a sense of obligations for members to serve the interest of the in-group and the fulfillment of duty to unite the community. Such sense of parochial altruism initially evolved as a response to the adaptive challenges of forming a cohesive coalition to compete for resources, territory, and powers with other groups of people. The original birthplace of this morality is the kin relationships that are based on shared blood and marriage, but loyalty has been extended to more impersonal, imagined communities, such as cities, regions, cultures, or nations (Haidt and Graham, 2006). In the name of loyalty, people tend to limit the scope of individualizing foundations toward outsiders (Bernhard et al., 2006; Fehr et al., 2008), and they are willing to sacrifice their own resources for their group while ignoring harm and injustice inflicted on outsiders (Baron et al., 2013).

Loyalty can be interpreted as a psychological ability to depersonalize the self and to integrate into the group by categorizing individuals, exemplifying the group, and adhering to values and norms that embody the group’s identity (Ashforth and Mael, 1989). Triggered by signs of in-group and out-group boundaries, Loyalty elicits emotions such as group pride, shame, and anger, and creates a calling to sacrifice for the in-group and to be viligient toward the out-group. It moralizes public service by enabling individuals to derive utilities from activities and objects that are in support of the group’s identity. The vast donation to rebuild the Notre Dame de Paris serves as a good example of how an impersonal object can become a calling to contribute, as French President Emmanuel Macron wrote: “Notre Dame of Paris in flames. Emotion for a whole nation.”^4^ Such social identification increases the homogeneity in beliefs, attitudes, and behaviors, which in turn engenders a shared sense of belonging, sustaining intra-group cooperation and group-based altruism, even in the absence of strong leadership. Therefore, Loyalty creates a patriotic logic of appropriateness: those driven by Loyalty see themselves as guardians of the community or the nation, exemplifying the group identity and fostering social cohesion and security (Ashforth and Mael, 1989; Perry, 2000; Boyd et al., 2018).

Loyalty-driven PSM creates a sense of community or citizenship, motivating people to take obligations to compatriots and to engage in activities that are congruent to this identity (citizenship behavior) (Mason, 1997). However, grouped-based altruism also implies that the needs of compatriots take precedence over the needs of outsiders. In the public sector, Loyalty is most emphasized in military, police and fire service to instill emotional commitment to public security service and stimulate courage to make self-sacrifice (Ewin, 1990; Connor et al., 2019). For instance, Braender and Andersen (2013) find that soldiers’ PSM and commitment to public values increase after deployment in Afghanistan because soldiers mutually reinforce their shared sense of service duty and professional identity during deployment. Outside of public organizations, the sense of community is an essential catalyst for voluntary participation in local action and neighborhood development (Chavis and Wandersman, 2002).

Proposition 4: Triggered by signs of in-group and out-group boundaries, Loyalty moralizes public service through increased attention to membership boundaries, elicits the sense of community and the obligations to promote social cohesion and security, and stimulates citizenship behavior.

In the evolution of humankind, *Sanctity* was shaped by the psychology of disgust and elevation. Sanctity underlies the spiritual purity of striving to live in an elevated, less carnal, and more noble way (Haidt, 2012). Sanctity initially emerged as a response to the adaptive challenges of avoiding disease transmission because of living in larger and denser groups. Our ancestors developed an effective “behavioral immune system” to detect infectious pathogens, but the system also responded to “an overly general set of superficial cues” that pose no actual threat of disease transmission, but can still provoke aversive feelings and responses (Schaller and Park, 2011). Sanctity stresses the priority of the soul over the body, and imposes strict rules on the “pure” use of the body (Giner-Sorolla et al., 2012). People feel disgusted and repelled when witnessing behaviors viewed as degrading or inhuman, whereas they feel uplifted and elevated when witnessing acts of moral beauty and perfection (Haidt, 2000; Haidt and Morris, 2009). Therefore, feelings of elevation and disgust can foster a desire for close affirmation of good deed doers and strong defense against a morally reproachable other.

Sanctity diminishes the self and generates a sense of purpose in life by creating the notion of spirituality or self-transcendence, the feeling of being connected to or monitored by a sacred, non-materialistic whole such as God, the natural environment, or humanity. It directs one’s attention to a meaning or purpose that is higher, more important than the one’s usual ‘banal’ concerns (Haidt and Morris, 2009). Spirituality has been found in the management literature to improve employees’ performance and organizational effectiveness by providing a sense of meaning and interconnectedness for employees to feel passionate and abundant (Karakas, 2010).

Sanctity-driven PSM moralizes public service as a noble calling that attracts a particular type of individuals who seek interconnection with the community or humanity as a whole,^5^ and such interpretation of public service can improve the commitment and competency of public employees (Perry, 1996; Pattakos, 2004; Houston and Cartwright, 2007; Ferguson and Milliman, 2008). Sanctity-driven PSM engenders a sainted logic of appropriateness that motivates to achieve self-transcendence: those driven by Sanctity see themselves guardians of collective and transcendent purposes, framing public service as “a unique, humanistic process of spiritual connection and enlightenment that helps groups achieve their collective and often transcendent aims” (Pattakos, 2004, p. 107). For instance, Sanctity moralizes pro-environment behavior by connecting the self to the natural world and creating an elevated feeling toward animals, plants, or other aspects of nature (Moreton et al., 2019).

In public organizations, public employees are expected to preserve the sanctity of public service by keeping public service “unspotted from the dirty political world” (Denhardt, 1988, p. 58), and by restraining themselves from the abuse of power and corruption that are deemed to be detrimental to society. Attaching public service with a spiritual connection is also found to help law enforcement officials to appreciate their routine duties that require emotional labor toward negative feelings from clients (Dutelle and Taylor, 2017, pp. 46–48). Without such moralization, officials working in emotional labor-intensive functions (such as healthcare, education, law, and social work) tend to become cynical and unmotivated.

Proposition 5: Triggered by signs of piety and degradation, Sanctity moralizes public service through increased attention to meaning and connection to higher purpose, elicits the sense of spirituality that connects the self to a collective and transcendent purpose, and stimulates self-transcendence behavior.

## Boundary Conditions for Moralizing Public Service

Public Service Motivation literature has documented the cultural variation in the meanings and connotations of subdimensions of PSM, which may result in different patterns of PSM across countries (Vandenabeele and van de Walle, 2008; Kim et al., 2012; van der Wal, 2015). By including a broader range of moral domains and encompassing the constellations of each moral values and social practices, this theoretical study demonstrates that different moral foundations could be useful to disaggregate the psychological antecedences of PSM, and to explore the cultural, institutional, and individual variations in the meaning of PSM. In this section, by way of illustration, we will provide two propositions regarding the boundary conditions of moralizing public service, which can be helpful in developing hypotheses regarding moderating and meditating influences.

As mentioned, different cultures and organizations may employ their own configurations of moral foundations to construct moral codes, value systems, and social norms that specify desirable and inappropriate behaviors. In other words, cultural norms may tweak our moral mind and cognition process to help people adapt to a particular social environment (see McNamara et al., 2019). For instance, although individualizing moral foundations are widely shared across cultures, collective cultures tend to rely on binding foundations more than do individualistic cultures (Vauclair et al., 2014). Individualistic cultures could even devalue binding foundations in constructing public service morality, making binding foundations to be negatively associated with PSM. As a result, the configuration of moral foundations may differ in constructing PSM across cultures, geographies, and languages, even though individualizing foundations are more universally endorsed (e.g., Wheeler et al., 2019).

For instance, religion, as a salient cultural phenomenon, has been found to influence the concept of PSM (Vandenabeele et al., 2004) and MFs (Johnson et al., 2016). Religion provides triggers of moral foundations that can also be associated with stimuli regarding public service. Catholic morality such as deliverance (related to Sanctity) and obedience (Authority) is institutionalized within the public service in a Catholic country such as France, while Protestant morality such as work ethic and egalitarianism (related to Fairness) is rooted in the public value in the Netherlands (Houston et al., 2008). Similarly, Kim (2009) investigates PSM in Korea and suggests that in a Confucian-oriented society, people tend to respect and honor governments’ bureaucrats with a higher social status because of their superior benevolence and administrative ability, which implies the important role of Authority in shaping the commitment to and rationale regarding public service in East Asian countries.

Proposition 6: The saliance of moral principals in a culture influences an individual’s social cognition process of PSM, implying that the configuration of moral foundations differs across cultures in moralizing public service.

Public organizations provide sufficient opportunities for individuals to serve the public and satisfy their public service motives. Hence, public employees may be self-selected into public employment. However, an employee’s moral identity may not be consistent with the organization’s mission and value propositions, or employees may find the management culture or the ways of implementing public decisions contradictory to their conceptions of ideal public service (Wright and Pandey, 2008). Person-organization value congruence, therefore, can influence an individual’s tendency to moralize public service in public organization. For instance, Fehr et al. (2015) develop a model of moralized leadership, arguing that the follower will moralize leaders’ behavior that is consistent with the moral foundations of the follower and the organizational culture. A misfit between a public employer’s moral constellation and the institutional environment may lead to a moral dilemma and inhibit the moralization of certain public service behaviors that leaders and the organization intend to promote (O’Reilly and Chatman, 1986; Krogsgaard et al., 2014; Jensen et al., 2019).

Proposition 7: Person-organization moral congruence promotes the moralization of public service and moderates the effect of MFs on PSM behaviors.

## Discussion

### Theoretical and Practical Contribution

This study theoretically investigates the social cognition process associated with prosocial motivation, and links a range of moral foundations to Public Service Motivation (PSM) and behavioral consequences. In so doing, we take Moral Foundation Theory (MFT) to develop a theory of a causal PSM map, as such a casual map is still underdeveloped in the current public administration literature. In particular, how PSM is distinguishable from altruism and related concepts is still contested (Bozeman and Su, 2015). Specifically, we contribute to PSM theory by emphasizing the underlying cognition process and by providing microfoundations for a broad range of PSM-related behaviors. We do so by identifying PSM’s trigger for moral intuitions, the resulting locus of attention and belief elicitation, the representation of the emotional state (the logic of appropriateness), and specific types of motivated behaviors. Although our study focuses on explaining the moralization of public service, the proposed social cognition process framework can be used to analyze other social and organizational behaviors as well (see Weaver et al., 2014; Fehr et al., 2015).

By including moral concerns beyond empathy and social justice, we avoid making normative assumptions regarding the moral contents of PSM. This instead allows us to adopt a more pluralistic view toward PSM. The pluralistic approach is particularly important to further build PSM research internationally. First, the meaning and scaling of PSM dimensions are found to differ across different cultures and languages, even though the PSM measurement is confined to democratic and right-based concerns (Kim et al., 2012). Second, current PSM theory has been found to be “WEIRD” and thus problematic in explaining the motivational behavior and organizational dynamic in non-Western contexts, even in a democratic country such as South Korea (Kim, 2009; van der Wal, 2015). Third and lastly, adopting a pluralistic conceptualization of PSM not only helps to internationalize PSM research, but also allows us to explore diverse altruistic motives that stimulate public service, improving the understanding of basic psychological needs behind PSM.

Beyond this theoretical contribution, the study also suggests potentially important practical implications regarding the use of PSM to stimulate prosocial behaviors. Individuals can hold different conceptions of PSM by associating different social experiences and life events with their innate morality. Public organizations should consider individuals’ innate morality and its behavioral consequences when motivating specific types of prosocial behavior. The social cognition process of PSM as spelled out in this study provides a framework for public organizations to think about ways to utilize different configurations of moral foundations, providing individual employees with relevant codes of conduct, social identities, and motivational vocabularies.

### Limitation and Future Research Directions

We present several propositions that can be developed into testable hypotheses. Since MFT and PSM have both developed validated measurements of five moral foundations (Graham et al., 2009) and four PSM-subdimensions (Kim et al., 2012), respectively, the first step is to empirically investigate their relationships and behavioral implications through representative survey data across different countries. Also, the so-called Moral Foundations Dictionary, developed by Graham et al. (2009), can be used to conduct textual analysis on organizational documents to measure moral configurations across different public organizations or departments, and subsequently test person-organization moral congruence and its effect on PSM. For instance, the public security and safety sector, such as the police and military, may emphasize Loyalty when moralizing public service, while the health care and education sectors may rely on Care and Sanctity to promote public service.

Also, it will be helpful to explore how the Western ideas of public values are reconciled with religious and social traditions in non-Western societies and semi-democracies to exhibit PSM. Even within Western societies, public institutions in cosmopolitan cities and provincial towns could rely on different sets of moral foundations to moralize public service. Exploring these differences, within and across countries, can help PSM theory to become more applicable in explaining public service beyond Western democratic societies, and shed some light on how to manage “culture wars” within the public domain in a more and more ideologically polarized society (Rosenbloom, 2010; for cross-culture adjustment in workplace, see also Giorgi et al., 2020).

Our study has explored moral intuitions and social stimuli behind various PSM-related behaviors. However, when relying on certain moral concerns to construct their motivations and preferences regarding public service, individuals may inevitably bring their “biases” or worldviews into public administration^6^. Prokop and Tepe (2020) find evidence in a lab experiment that individuals who are attracted to public service tend to enforce a Fairness norm through unnecessarily excessive sanctions. By understanding the cognitive bias that moral foundations entail, such as punitive behavior, blind loyalty, and rule-bending, future studies could contribute to a recent thread of research on the “dark side” of PSM (Schott and Ritz, 2017), and provide practical implications as to how to manage moralized behaviors. For instance, the willingness to blow the whistle is shown to be predicted by a tradeoff between Fairness and Loyalty (Waytz et al., 2013). Public institutions that intend to promote whistle-blowing behavior, therefore, can embed Fairness-relevant stimuli and avoid relying on Loyalty to associate with PSM.

Finally, our study argues that PSM involves a cognition process that links automatic emotional responses with explicit knowledge of public service to construct a representation of the social world. We focus our discussion on how the concept of public service is associated with moral intuitions and become moralized in public institutions. However, how deliberation can override or reappraise automatic intuitions is beyond the scope of this paper, but important to enquire. For instance, on the one hand, Stazyk and Davis (2015) observe that public employees who lack advanced professional degrees are more likely to favor personal intuitions over externally derived obligations in the context of decision-making as PSM increases. So, professional education may enhance the cognitive ability to reappraise features of the situations and regulate emotional reactions. On the other hand, research has shown that individualizing foundations require abstract and analytic thinking when making moral decisions (Napier and Luguri, 2013; Pennycook et al., 2014; Yilmaz and Saribay, 2017). People also tend to give consequentialist, non-emotional justification (based on the outcomes or consequences of actions) to Care- and Fairness-related moral decisions (Wheeler and Laham, 2016). Therefore, Care- and Fairness-driven PSM may increase the likelihood to make public decisions with consequentialist reasoning, such as cost-benefit and welfare analysis. Future studies could investigate the interaction between intuitive and deliberative processes, which will be helpful to uncover the role of reasoning in reappraising moral intuitions and develop interventions to mitigate the dark side of PSM.

## Conclusion

Public Service Motivation is a motivational model built on a logic of appropriateness: Self-identity can interact with contextual stimuli, and can define individuals’ perception of situations they face in their organization (Perry, 2000; Vandenabeele, 2007). MFT delineates five moral intuitions that humans have evolved since our ancestors faced a diverse set of longstanding adaptive challenges to organize social lives. We illustrate how PSM can be constructed through the lens of cognitive science, and then show how moral foundations can disaggregate the construct of PSM. As a multi-dimensional construct, PSM is related to a pluralistic set of moral concerns that people can associate with their life experiences and social environment in order to establish a sense of public morality. Public values endorsed in the modern, democratic institutions are mostly consistent with the individualizing foundations of Care and Fairness. However, people who feel motivated to contribute to the public good can regard public service not merely as compassionate and just, but also as respectable, patriotic, and transcendent. By taking the full range of moral intuitions in accounts, MFT provides psychological microfoundations in explaining a broad range of PSM behaviors.

## Author Contributions

TW developed the theoretical model and hypotheses and wrote the manuscript. AW and FH supervised the work, provided comments on different versions of the manuscript, and edited the final manuscript. All authors contributed to the article and approved the submitted version.

## Conflict of Interest

The authors declare that the research was conducted in the absence of any commercial or financial relationships that could be construed as a potential conflict of interest.

## Footnotes

1 Henley, J. (September, 03, 2015). Britons rally to help people fleeing war and terror in Middle East. *The Guardian*. Retrieved from https://www.theguardian.com/uk-news/2015/sep/03/britons-rally-to-help-people-fleeing-war-and-terror-in-middle-east.

2 More precisely, ingredients to construct a mental representation (or emotional feeling) in a cognitive workspace includes sensory information about the stimulus and the environment, the activated survival circuit (which identifies opportunities or threats), information about the elicited generalized arousal, body feedback from innate responses, and long-term memories about the stimulus and the resulting state (LeDoux, 2012, p. 665).

3 Fukuyama (2018) terms “Megalothymia” as the need to be recognized as superior to others and suggests such desire to be inherent to every human being. Megalothymia does not just reflect the vanity of the ambitious; it constitutes the just deserts of the virtuous. Some people need to be valued at a lower rate than others because of their superior virtues or knowledge.

4 Emmanuel Macron (@EmmanuelMacron), “Notre-Dame de Paris en proie aux flammes. Émotion de toute une nation.” Twitter, April 15, 2019 8:05 p.m., https://twitter.com/EmmanuelMacron/status/1117851407644684288.

5 For example, Bruce (2000) finds in a survey that 60% of public sector employees feel public service as a spiritual calling, and 48% see their work as a part of their spiritual path.

6 As an extreme example, totalitarian regimes such as that of the Nazis rely heavily on binding foundations to construct a public service identity that is normatively evil but felt righteous among officials and followers. Acknowledging the positive existence of binding foundations does not normatively recognize or justify their biases. Instead, it allows us to investigate their dark side, and to identify limitations and dangers of a behavior’s moralization.
